# TROP-2 is widely expressed in vulvar squamous cell carcinoma and represents a potential new therapeutic target

**DOI:** 10.1007/s00432-023-04761-8

**Published:** 2023-04-17

**Authors:** Mateja Condic, Eva K. Egger, Niklas Klümper, Glen Kristiansen, Alexander Mustea, Thore Thiesler, Damian J. Ralser

**Affiliations:** 1 Department of Gynecology and Gynecological Oncology, University Hospital Bonn, Bonn, Germany; 2 Department of Urology and Pediatric Urology, University Hospital Bonn, Bonn, Germany; 3 Institute of Pathology, University Hospital Bonn, Bonn, Germany

**Keywords:** Vulvar squamous cell carcinoma, TROP-2, Antibody-drug conjugate, HPV

## Abstract

**Purpose:**

Vulvar squamous cell carcinoma (VSCC) is a rare malignancy of the female genital tract with increasing incidence rates. Etiologically, HPV-dependent and HPV-independent VSCC are distinguished. Surgical treatment and/or radiotherapy represent the therapeutic mainstay for localized disease. For recurrent or metastatic VSCC, treatment options are limited. Research has identified trophoblast cell surface antigen 2 (TROP-2) to be broadly expressed across different tumor entities. The aim of the present study was to systematically investigate the expression of TROP-2 in VSCC.

**Methods:**

TROP-2 protein expression was investigated by immunohistochemistry in a cohort comprising *n* = 103 patients with primary VSCC. A four-tier scoring system (0: no staining, 1 + : low staining, 2 + : moderate staining, 3 + : high staining) was applied for quantification of protein expression. For further analyses, two groups (low TROP-2 expression: 0/1 + ; high TROP-2 expression: 2 + /3 +) were generated. The entire study cohort, as well as HPV-dependent and HPV-independent VSCC were considered separately.

**Results:**

In the entire VSCC study cohort, TROP-2 expression was present in 97.1% of all cases (*n* = 100) with 74.8% displaying high TROP-2 expression (2 + /3 +). Only 2.9% of tumors showed absent TROP-2 expression. Of note, all HPV-dependent VSCC (*n* = 18) demonstrated high TROP-2 expression (2 + /3 +). In the subgroup of HPV-independent VSCC (*n* = 70), high TROP-2 expression was associated with favorable clinical outcomes based on log rank test and univariate cox analysis.

**Conclusion:**

TROP-2 protein expression is of prognostic value in HPV-independent VSCC. The broad expression of TROP-2 in VSCC indicates the TROP-2 directed ADC Sacituzumab govitecan as a potential new therapeutic strategy for VSCC patients.

## Introduction

Vulvar cancer (VC) is the fourth most common genital tumor in women, accounting for 3% of all gynecological cancers worldwide (Sankaranarayanan and Ferlay 2006). Albeit VC is considered a rare tumor entity, its incidence has increased over the recent decade by 20%. This is mainly attributed to the increased prevalence of human papillomavirus (HPV) infections and the overall demographic aging of the population (Schuurman et al. 2013; Kang et al. 2017).

The predominant histological VC-subtype is vulvar squamous cell carcinoma (VSCC). Etiologically, HPV-dependent VSCC, which account for one-third of the cases (Zhang et al. 2018), and HPV-independent VSCC, which develop on the basis of *lichen sclerosus et atrophicus* (Bleeker et al. 2016), are distinguished. Besides the different etiology, the prognosis of these two VSCC subtypes also differs with HPV-independent VSCC exhibiting a comparably worse overall prognosis. The therapeutic mainstay for localized VSCC is surgical resection of the tumor with bilateral sentinel inguinofemoral lymphonodectomy or systematic bilateral inguinofemoral lymphonodectomy, followed by radiotherapy if appropriate risk factors are present. Whereas localized VSCC has an 5-year overall survival (OS) rate of over 85% (Schnürch et al. 2015), recurrent, advanced or metastatic disease has a limited 5-year OS rate of only 15–50% (Nooij et al. 2016). This is predominantly attributed to the limited therapeutic options. Hence, for this population, there is an unmet need for new therapeutic options (Clancy et al. 2016).

Trophoblast cell surface antigen 2 (TROP-2) was initially described as a transmembrane protein on the surface of trophoblast cells (Ripani et al. 1998). In the last decade, research has shown that TROP-2 plays an essential role in regulatory processes of carcinogenesis and cancer progression thereby accomplishing the function of an oncogene. Recently, however, there is growing scientific evidence that TROP-2 is capable to promote both, tumor progression and tumor suppression, depending on the cell context and localization (Zhang et al. 2014; Zeng et al. 2016). The precise pathomechanism for these conflicting functions remains to be elucidated. Regardless of its function, TROP-2 represents a promising therapeutic target, especially since TROP-2 is widely overexpressed across different tumor entities (Goldenberg et al. 2018). Sacituzumab govitecan (SG) is an antibody–drug conjugate (ADC) consisting of an antibody targeting TROP-2 that is linked to the cytotoxic payload SN-38, a topoisomerase I inhibitor. SG has recently been approved for the treatment of metastatic triple-negative breast cancer and is considered for further indications with plethora of clinical trials ongoing (Bardia et al. 2019). However, up to now, SG is not considered in the treatment of VSCC. For cervical carcinoma (CC), a gynecological malignancy with etiological resemblance to VSCC, research has shown that gradual loss of TROP-2 plays a role in the progression of intraepithelial neoplasia to invasive carcinoma and exhibits tumor suppressive functions (Wang et al. 2014; Sin et al. 2019). Further, there is preclinical evidence that therapy directed against TROP-2 is effective in CC (Zeybek et al. 2020). To the best of our knowledge, there are no data on TROP-2 expression in VSCC. The aim of the present study was (i) to investigate the expression of TROP-2 in VSCC, (ii) to determine its impact on clinical outcomes, and (iii) to evaluate its potential to serve as a therapeutic target.

## Methods

### Patients and specimens

The retrospective single-center study cohort included *n* = 103 patients with primary VSCC treated at the University Hospital Bonn between 2002 and 2017. Tissue was obtained from biopsies or surgical specimens that were collected within the framework of the Biobank initiative of the University Hospital Bonn. The Ethics Committee of the Medical Faculty of the University of Bonn approved the study (vote: 208/21).

Clinicopathological characteristics of the entire cohort, and the HPV-independent and the HPV-dependent sub- cohorts were obtained from a clinical database. Details are depicted in Table 1. Histopathological diagnosis was based on the World Health Organization (WHO) criteria. Pathological grading was determined applying the International Federation of Gynecology and Obstetrics (FIGO) that was revised in 2010. Tumor stage was classified according to the 7th TNM classification of the Union for International Cancer Control (UICC).

**Table 1:** Clinicopathological characteristics of the entire VSCC cohort, HPV-independent cohort, and HPV-dependent cohort

Clinicopathological parameters	All (*n* = 103)	HPV-independent (*n* = 70)	HPV-dependent (*n* = 18)
Age (years)			
Mean (± SD)	64.8 ± 14.4	65.7 ± 14.0	56.9 ± 14.1
Min–max	25–93	33–93	25–77
Overall survival (months)			
Mean *(*± *SD)*	53.0 ± 43.5	57.6 ± 46.0	47.9 ± 41.3
Median	44.0	55.5	46.0
TNM classification			
T1	80 (77.7%)	56 (80.0%)	11 (61.1%)
T2	16 (15.5%)	12 (17.2%)	4 (22.2%)
T3	3 (2.9%)	1 (1.4%)	2 (11.1%)
Tx	4 (3.9%)	1 (1.4%)	1 (5.6%)
N0	37 (35.9%)	29 (41.4%)	2 (11.2%)
N1	10 (9.7%)	6 (8.6%)	4 (22.2%)
N2	20 (19.4%)	16 (22.9%)	4 (22.2%)
N3	1 (1.0%)	0	0
Nx	35 (34.0%)	19 (27.1%)	8 (44.4%)
Grading			
G1	8 (7.7%)	4 (5.7%)	1 (5.6%)
G2	66 (64.3%)	46 (65.7%)	12 (66.7%)
G3	26 (25.1%)	18 (25.7%)	4 (22.2%)
Not determined	3 (2.9%)	2 (2.9%)	1 (5.5%)
HPV-subtypes			
16			14 (77.8%)
33			3 (16.7%)
33 + 16			1 (5.5%)

For *n* = 15 patients, no HPV status was available

*SD* standard deviation

### Tissue microarray (TMA) construction

The TMA was generated from formalin-fixed paraffin (FFPE)-embedded VSCC tissue specimens. Representative tumor areas in hematoxylin and eosin (HE) stained sections were identified. 1 mm core biopsies (0.785 mm^2^) were taken from the selected cancer areal and arranged in TMA blocks.

### HPV analysis

Determination of HPV subtypes was performed applying the HPV Type 3.5 LCD-Array Kit (Chipron, Germany) according to the manufacturer’s instructions and as described previously (Hecking et al. 2017).

### Immunohistochemistry

Immunostaining of TROP-2 was performed on VSCC- TMAs using an automated staining system (BenchMark ULTRA; Ventana Medical Systems, Tucson, AZ, USA) which performed deparaffinization, pretreatment with cell conditioning buffer (CC1 buffer, pH8), and incubation with the primary TROP-2 antibody (1:1500, Enzo Life Sciences Inc, Farmingdale NY, USA, Clon-01 IgG1, mouse) at 4 °C overnight. Signal detection was obtained with the UltraView DAB IHC Detection Kit (Ventana Medical Systems, Tucson, AZ, USA). Analysis of immunostained cells was carried out with an Olympus BX51 microscope and the Panoramic Viewer 3DHistech. All staining intensities were evaluated separately by MC and DJR. TT was consulted as a board-certified gynecopathologist in case of discordance between these two investigators. Staining intensities were categorized in a four-tier scoring system (0: no staining, 1 + : low staining, 2 + : moderate staining, 3 + : high staining). Two groups, low (0/1 +) and high (2 + /3 +) expression, were generated for survival analysis.

### Statistical analysis

Kaplan–Meier survival analyses, log-rank tests and univariate cox analyses were conducted to compare OS between the two groups (low vs. high expression of TROP-2) for the entire study cohort and the HPV-independent subgroup. Significance threshold was considered at a *p*-value of < 0.05. Statistical analysis was performed with the Statistical Package for the Social Sciences (SPSS ^®^) version 29 (SPSS Inc., IBM Corp.) and the GraphPad Prism software (GraphPad software).

## Results

### TROP-2 is frequently expressed in VSCC

**Fig. 1:**
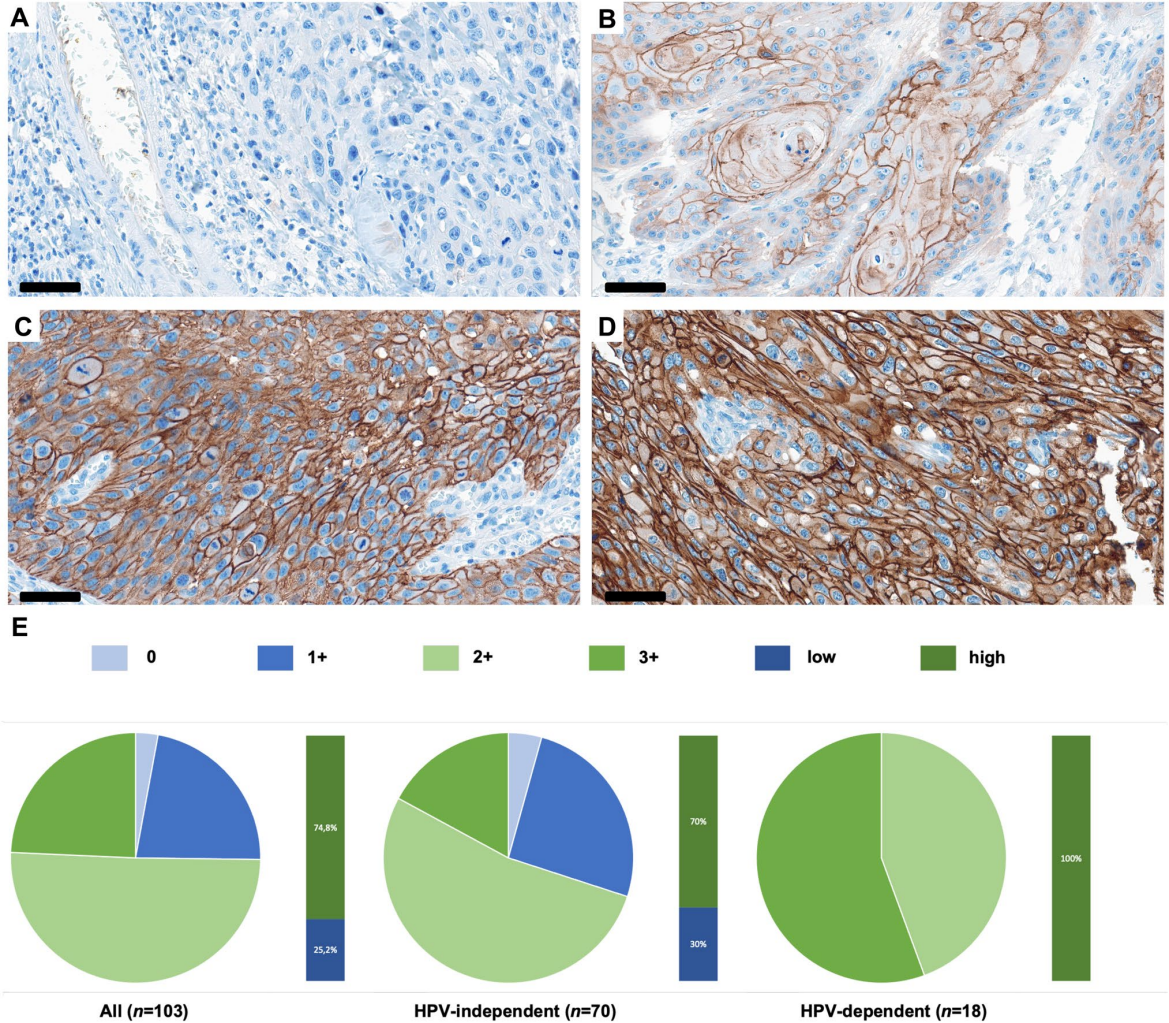
Representative histology sections showing absent (0; A), low (1 + ; B), moderate (2 + , C), and high (3 + , D) TROP-2 protein expression. (E) Pie charts illustrating the distribution of expression intensities in the entire VSCC cohort, the HPV-independent VSCC cohort, and the HPV-dependent VSCC cohort. Bar graphs showing differentiation into low (0, 1 +) and high (2, 3 +) TROP-2 protein expression. Scale bar = 60 µm

Immunohistochemical staining of TROP-2 revealed strong membranous expression. In the entire study cohort, TROP-2 expression was present in 97.1% of all cases (*n* = 100). Only 2.9% of tumors showed absent TROP-2 expression (0, Fig. 1A). Low expression (1 + , Fig. 1B) was detected in 22.3% of the cases. The majority of all samples (74.8%, *n* = 77) exhibit moderate (2 + , Fig. 1C) to high (3 + , Fig. 1D) TROP-2 expression. The distribution of the different expression intensities across the entire study cohort and the individual sub-cohorts (HPV-independent/dependent tumors) is depicted in Fig. 1E.

### HPV-dependent VSCC displays exclusively moderate to high TROP-2 expression

The sub-cohort of HPV-dependent VSCC comprised *n* = 18 cases. *n* = 70 cases were HPV negative and for *n* = 15 cases, HPV testing was inconclusive. Whereas TROP-2 expression covered the whole expression intensity spectrum in the HPV-independent VSCC population, HPV-dependent VSCC exhibit exclusively high TROP-2 membranous staining pattern (Fig. 1 E).

### High expression of TROP-2 correlates with favorable outcomes in HPV-independent tumors

**Fig. 2:**
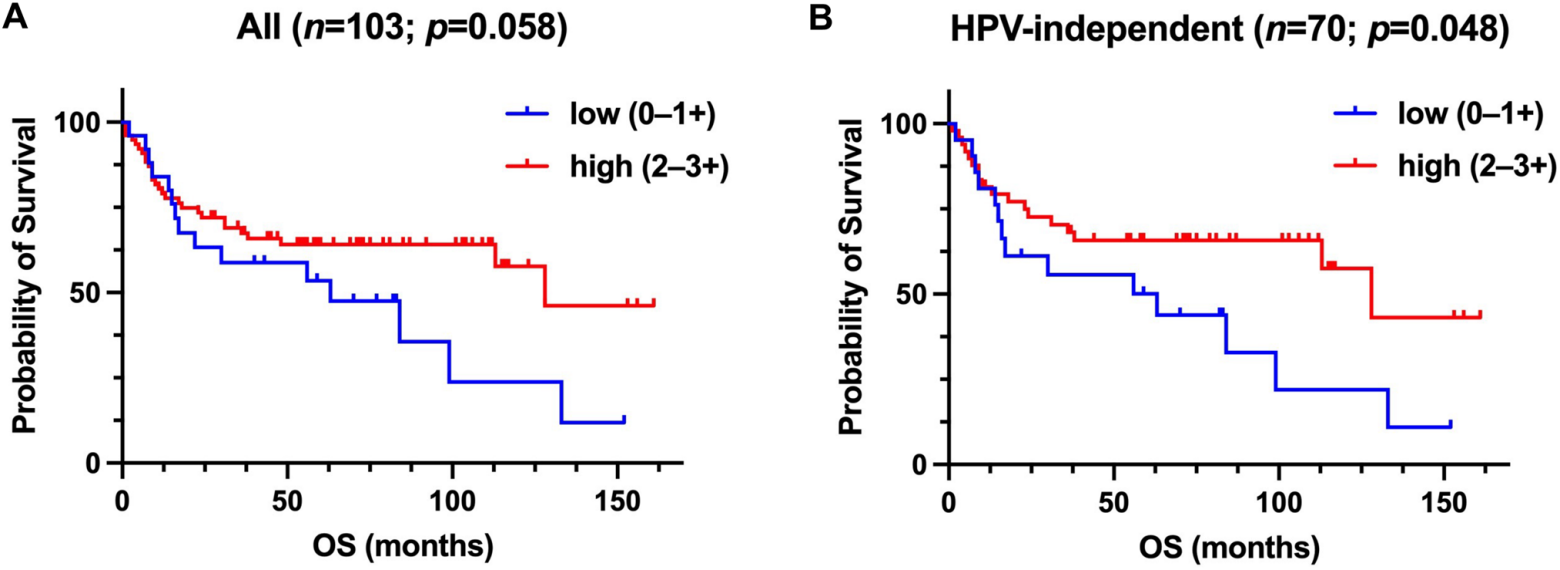
Kaplan–Meier estimates show a trend towards a shorter overall survival (OS; *p* = 0.058) in patients with low expression of TROP-2 in the entire VSCC cohort (A). In HPV-independent VSCC, high TROP-2 expression was linked to favorable OS (*p* = 0.048; B). *P*-values for the group comparison (low vs., high expression) are based on log-rank tests, significance threshold *p* < 0.5

In the entire study cohort, the median OS was 44 months. In the entire cohort, TROP-2 expression was not significantly associated with OS (Fig. 2A, log rank *p* = 0.058). However, there was a trend indicating that tumors with high TROP-2 expression levels exhibit a better prognosis. In the subgroup of HPV-independent VSCC, TROP-2 expression was significantly linked to OS. In particular, low TROP-2 expression (0/1 +) was linked to a shorter OS compared to high TROP-2 expression (2 + /3 + ; *p(*log) = 0.048*;* HR 0.5 (95%CI: 0.248–1.009), *p*(cox) = 0.05; Fig. 2B*).* TROP-2 protein expression was not associated with known clinicopathological prognostic parameters like nodal stage and histomorphological grading.

## Discussion

The present study is, to the best of our knowledge, the first systematic investigation of TROP-2 protein expression in VSCC tissue. TROP-2 was found to be broadly expressed in VSCC which is highly relevant from a therapeutic point of view as there is an approved TROP-2 directed therapy with the ADC SG available and further TROP-2 ADC are currently in preclinical and clinical testing. Further, we provide evidence for the prognostic value of TROP-2 protein expression in HPV-independent VSCC. In our HPV-independent VSCC study cohort, higher TROP-2 expression levels were linked to favorable clinical outcomes.

TROP-2 was previously classified as an oncogene and high TROP-2 expression levels were linked to poor prognosis across various tumor entities including breast cancer (Lin et al. 2014), prostate cancer (Trerotola et al. 2013), and colon cancer (Zhao et al. 2015). However, contrasting tumor suppressive properties for TROP-2 have been described in head and neck carcinoma and lung carcinoma (Zhang et al. 2014; Erber et al. 2021). A recent study showed epithelial-mesenchymal transition of keratinocytes and consecutive skin tumor formation in TROP-2 knockout (ARF^−/−^ C57BL/6) mice (Wang et al. 2011), suggesting that in the context of squamous cell carcinoma, TROP-2 fulfills the function of a tumor suppressor. Another study investigated TROP-2 expression in normal tissue and in squamous cell carcinoma (SCC) of the cervix, esophagus, and head and neck. The authors found that a gradual loss of TROP-2 was associated with a stepwise progression from precursor lesions to invasive SCC. Additionally, TROP-2 expression affected treatment response (Wang et al. 2014). Further evidence for the tumor suppressive function of TROP-2 is provided by *Sin *et al*.* who demonstrated that TROP-2 reduced oncogenicity of CC cells (Sin et al. 2019). Congruent with these results, TROP-2 was broadly expressed in our VSCC-cohort and low expression levels were associated with unfavorable outcomes. Thus, TROP-2 could potentially exert a tumor suppressive function in VSCC, with loss of TROP-2 appearing as a sign of increasing dedifferentiation in aggressive VSCC. Of particular note, we found that all HPV-dependent VSCC samples showed moderate to high TROP-2 expression which indicates a connection between HPV-infection, TROP-2 expression and the etiology of HPV-dependent VSCC. Large proteomic analyses have identified more than 100 signaling pathways that are modulated by TROP-2, including the PI3K/AKT/mTOR pathway (Guerra et al. 2016). In the context of HPV-dependent SCC, alterations in the PI3K pathway are also described (Cochicho et al. 2022). The specific interaction between TROP-2 expression and HPV needs to be further elucidated. Besides the identification of TROP-2 protein expression to serve as a prognostic biomarker in HPV-independent VSCC, the broad expression of TROP-2 in VSCC provides a strong rationale to evaluate the anti TROP-2 ADC SG in clinical VSCC trials. Additional supporting data is available from Zeybek et al. (Zeybek et al. 2020), who found a moderate to strong TROP-2 staining in 95% of squamous cell cervical carcinoma by immunohistochemistry. TROP-2 positive cervical cancer cell lines were highly sensitive to SG in vitro (Zeybek et al. 2020). From a mechanistic point of view, tumorous TROP-2 expression represents the biological prerequisite for effective SG treatment. However, data from the approval relevant ASCENT study showed, that for triple negative breast cancer, the level of TROP-2 expression is not predictive for SG treatment response (Bardia et al. 2021). However, we have previously shown that target protein expression is relevant for the response to other ADCs, such as Enfortumab vedotin for patients with metastatic urothelial cancer (Klumper et al. 2022). Considering that in our study only 2.9% of VSCC were TROP-2 negative and 75% expressed moderate to high levels of TROP-2, is seems reasonable to propose SG as a novel targeted therapy option in VSCC. However, this needs to be further investigated in ideally biomarker-driven clinical trials.

## Conclusion

In this study, we demonstrate that TROP-2 is broadly expressed in VSCC. In our study cohort, HPV-dependent VSCC showed exclusively high expression levels of TROP-2, indicating a relationship between TROP-2 and HPV-dependent VSCC etiology. In HPV-independent VSCC, TROP-2 expression was of prognostic value and appears to have tumor suppressive function. Considering the broad expression of TROP-2 in VSCC, our study provides the rationale to evaluate TROP-2 directed therapeutic approaches in VSCC.

## Data Availability

The datasets generated and/or analyzed during the current study are available on request from the authors.
